# Utilization of digital prenatal services and management of depression and anxiety during pregnancy: A retrospective observational study

**DOI:** 10.3389/fdgth.2023.1152525

**Published:** 2023-03-30

**Authors:** Lily Rubin-Miller, Natalie Henrich, Alex Peahl, Christa Moss, Neel Shah, Hannah R. Jahnke

**Affiliations:** ^1^ Maven Clinic, New York, NY, United States; ^2^ Department of Obstetrics and Gynecology, University of Michigan, Ann Arbor, MI, United States; ^3^ Harvard Medical School, Boston, MA, United States; ^4^ Department of Obstetrics and Gynecology, Beth Israel Deaconess Medical Center, Boston, MA, United States

**Keywords:** pregnancy, mental health, anxiety, depression, digital resource, digital services, mental health management, health services

## Abstract

**Introduction:**

We examined how utilization of Maven, a digital healthcare platform that provides virtual prenatal services, is associated with improvements in perceived management of anxiety and depression during pregnancy, and how medical knowledge and support may influence this association.

**Materials and Methods:**

In this retrospective study we used adjusted logistic regression to examine the relationship between digital platform use in pregnancy and perceived mental health management, and how perceived management of mental health is affected by user-reported improvements in medical knowledge and feeling supported by the platform. Effects were evaluated separately among users with and without a mental health condition. Demographics, medical history, and mental health management were self-reported.

**Results:**

Of 5,659 users, 705 (12.5%) reported that Maven helped them manage anxiety and/or depression in the prenatal period. In adjusted models, users who read more articles, sent more messages to care advocates, or had more appointments with providers were more likely to report improved management of mental health in a dose-response manner (e.g., articles read: Q2 aOR 1.31 (95% CI 1.01–1.70), Q3 aOR 1.68 (95% CI 1.30–2.17), Q4 1.99 (95% CI 1.54–2.59)). Improvements in medical knowledge and high perceived support were both associated with better perceived mental health management. Results were similar in users with and without a mental health condition.

**Discussion:**

These results suggest that access to a diverse set of digital resources provides multiple pathways to managing depression and anxiety during pregnancy for those with and without a diagnosed mental health condition.

## Introduction

Mental health disorders during pregnancy are common, with 16.4% of pregnant people experiencing depression in the prenatal period (1). Rates of postpartum depression are higher in people who report depression before or during pregnancy (2). If left untreated, these conditions are associated with increased health care utilization, worse maternal and infant outcomes, and higher healthcare costs (3–5). While multiple national medical organizations, including the American College of Obstetricians and Gynecologists and the US Preventive Services Task Force, recommend that pregnant people be screened for depression at least once during pregnancy (2, 3), only 65% of pregnant people are screened as part of their routine prenatal care, and only 40% of those diagnosed receive adequate treatment (4, 6). This leaves many people, including those with less severe presentations, without the services and resources they need to support them throughout pregnancy. This gap is due in part to the siloed nature of health services, with obstetric care designed to address medical needs rather than emotional well-being (7).

In addition to the lack of screening in traditional care settings, a rising national shortage in mental health professionals contributes to the large number of mental health conditions that go undiagnosed and untreated. The Association of American Medical Colleges estimates that 150 million people currently live in areas with a shortage of mental health professionals, and that this gap will only grow in the next few years (8). There is an increasing demand for interventions that prevent, identify, and manage mental health conditions in ways that are both clinically impactful and cost effective (9).

Digital health platforms have a unique opportunity to offer solutions to these challenges in an efficient, convenient and patient-centered way by providing more accessible education, support, and mental health services. Multiple studies on the efficacy of mental health treatment delivered *via* telehealth have found that there is no difference in clinical outcomes when comparing digital services to face-to-face care (10–12). While mental health-focused digital health solutions have been found to benefit patients' mental health (13), this benefit eludes the many patients with undiagnosed or more mild mental health conditions, who do not seek mental health services.

Public health strategies, such as providing support, implementing collaborative care, and facilitating access to care, present promising avenues for addressing perinatal mental health (5). Digital platforms have an opportunity to effectively deliver these services, but the potential impact of digital platforms on management of depression and anxiety during pregnancy has not yet been well described. More research is needed to understand the potential benefits of digital health platforms that provide support and education as well as mental health resources to pregnant people, including those who might not seek out specialized care for mental health.

Maven is a comprehensive women's and family health digital platform that was developed to provide support services that supplement and complement routine prenatal care through digital services. Users receive free and unlimited access to Maven as an employer-sponsored health benefit through their own or their partner's employer. Within the digital platform, Maven offers educational content (articles, videos, and live classes), care coordination (through a dedicated care advocate), and provider services (virtual appointments and communication with a diverse team of providers). Maven services do not replace routine prenatal care.

**Figure 1:**
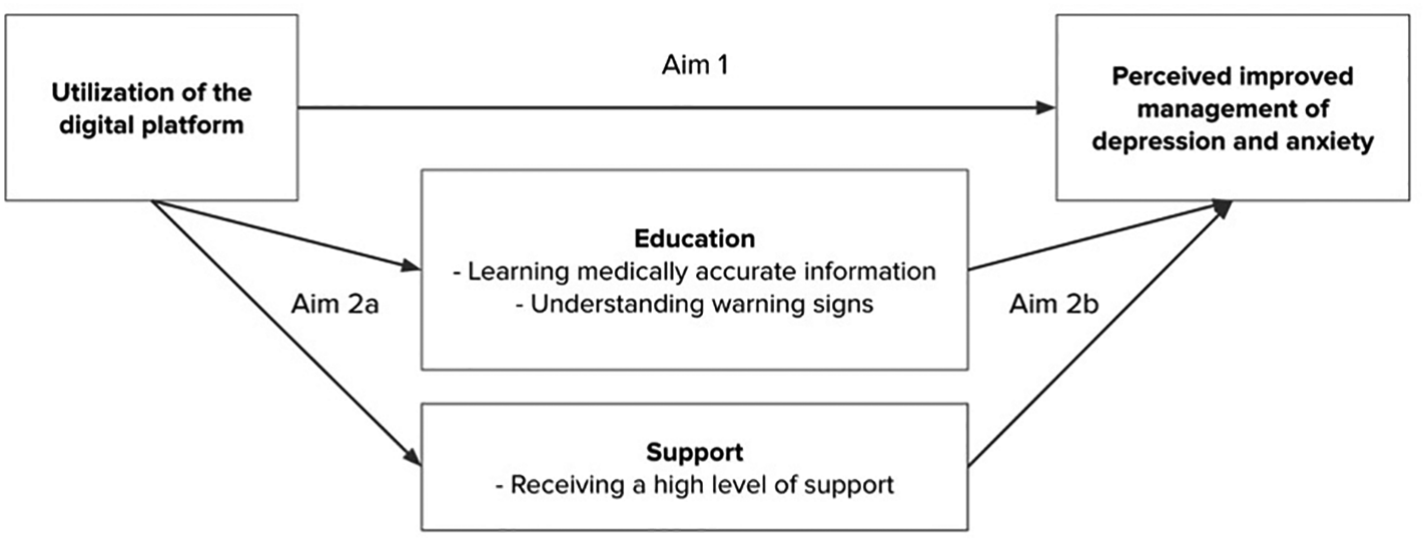
Conceptual model.

While there are many potential ways that digital services might help pregnant people manage their mental health, research has indicated that education and support are viable pathways to prevent and manage anxiety and depression (14). This study investigates how the use of services and resources on a digital health platform during pregnancy may help people with and without pre-existing mental health conditions better manage their mental health. We assess two aims: Aim 1) to assess *if* using digital services and resources has an effect on perceived improvements in management of depression and anxiety during pregnancy, and Aim 2) to examine *how* using digital services and resources may influence management of mental health through the pathways of education and support as the drivers of improvement (Aims 2a and 2b). A conceptual model demonstrates these pathways (Figure 1).

## Materials and methods

### Study design

In this retrospective cohort study, we examined the association between utilization of services and resources on a digital platform during pregnancy and user reports of improved management of anxiety or depression following delivery. Users were included if they enrolled from January 2020 to August 2022, completed a health survey at onboarding and one shortly following birth, and had delivered at the time of data analysis. Users without a valid ZIP code that could be mapped to the CDC's Social Vulnerability Index (SVI) were excluded. Upon creating an account for the digital platform, all users consented to the use of their de-identified data for scientific research. Utilization data were collected automatically through the platform and on self-report onboarding and post-delivery surveys. The study protocol was designated as exempt by the WCG Institutional Review Board.

### Digital platform services description and measurement

Data on utilization of services and resources were tracked within the platform and used as the primary exposure. Pregnancy trimester of enrollment was assessed categorically. Pregnancy trimester of enrollment was included as a predictor, and it was also a covariate in other models assessing the relationship between types of utilization and our outcome of interest. Categories of digital platform services included: (1) educational content, (2) care coordination, and (3) provider services.

1) *Educational content.* Educational content available through the platform included virtual classes, class recordings, and articles. Users have access to a variety of articles, classes, and videos related to pregnancy, birth, and the postpartum period. Example articles include, “How to take care of your mental health during pregnancy”, “Understanding your birth options”, and “What are the biggest feeding challenges for new moms?” In virtual classes users can engage with a health care provider in a group setting to learn and ask questions. Example classes include, “Self-advocacy during pregnancy, labor, and delivery” and “How to manage stress and anxiety”. Virtual classes are also recorded and uploaded to the digital health platform for users to watch asynchronously as video content. We assessed, in quartiles, the number of virtual classes attended, class recordings viewed, and articles read.
2) *Care coordination.* Care coordination encompasses interactions with the user's dedicated care advocate. We assessed user interaction with care advocates through messages in quartiles, and whether a user had at least one appointment with their care advocate.
3) *Provider services.* Provider services included interactions with healthcare providers in the digital health app through messages and appointments. Providers included obstetrician/gynecologists (OB/GYNs), midwives, doulas, mental health providers, nutritionists, wellness coaches, and others. We assessed messaging and appointments with any type of provider in quartiles. We also dichotomously assessed whether a user had at least one appointment in the platform with the following provider types; a mental health provider, an OB/GYN, a doula, and a midwife during their pregnancy, as these were the most utilized provider types.

### Self-reported measures

*User education*. Users were considered to have gained knowledge through interacting with the platform if they answered “Maven helped me learn medically accurate information” in response to “In what way(s) did Maven influence your experience?”, or answered yes to “Did Maven help you understand warning signs during pregnancy?” on the post-delivery survey. These outcomes were assessed separately.

*Perceived support.* Users reported their perceived support through the digital platform through the post-delivery survey question “On a scale of 1–5, how would you rate the support you received from Maven on your pregnancy journey?”. Responses were categorized into responses of 5 (excellent), 4 (very good), 1 to 3 (low support).

*Management of mental health*. Perceived management of mental health was based on users selecting “Maven helped me manage anxiety and/or depression” in response to the post-delivery survey question, “In what way(s) did Maven influence your experience?” This was the primary outcome.

*Demographic and medical information.* Demographic characteristics and medical conditions were self-reported. Race and ethnicity were assessed through the question, “How would you describe yourself?” with answer options of “American Indian or Alaska Native”, “Asian or Asian American”, “Black or African American”, “Hispanic, Latino, or Spanish origin”, “Native Hawaiian or Other Pacific Islander”, “White”, “I prefer not to say”. We consolidated multiracial, American Indian or Alaskan Native, and Native Hawaiian or Other Pacific Islander into an “Other” category due to small sample sizes.

We mapped the CDC's Social Vulnerability Index (SVI) (15), which provides a geospatial measure of a community's vulnerability, onto user ZIP codes using a 2020 weighted crosswalk from the US Department of Housing and Urban Development (16, 17). SVI was dichotomized into high and low, with high assessed as the most at-risk quintile nationally (>0.8) (18).

Medical conditions were assessed as a risk score, which was calculated as a count of key medical conditions including: chronic conditions (e.g., heart disease, diabetes, high blood pressure, blood disorder, thrombophilia, kidney disease, thyroid disease, autoimmune disease, HIV/AIDS, and obesity) and pregnancy conditions (e.g., cholestasis, fetal growth restriction, gestational hypertension, preeclampsia, gestational diabetes, and multiple gestation).

Mental health conditions were assessed as a dichotomous variable if a user reported any of the following: a history of anxiety, depression, perinatal mood disorder, or high pregnancy related anxiety. A history of anxiety or depression was assessed during the intake survey with the question, “Do any of these conditions apply to you or did they in the past?”, with the selection of “Anxiety / depression” from a list of conditions. Experience of perinatal mood disorder was assessed during either the intake or the post-delivery survey with the question “Have you experienced any of the following during this pregnancy?” with a selection of “Perinatal mood disorder”. Pregnancy-related anxiety was assessed on a 5-item Likert scale in response to “On a scale of 1–5, how anxious are you feeling about your pregnancy?”, with responses of 4 (“very”) or 5 (“extremely”) indicating the presence of pregnancy-related anxiety.

### Statistical analysis

We used descriptive analyses to assess users' demographics, risks, digital platform utilization during pregnancy, and education and support, stratified by presence of a mental health condition. In Table 1, chi-2 tests were used to assess the relationship between a categorical variable and the outcome (“Maven helped me manage anxiety/depression”) when all cell sizes were greater than 5. For categorical variables where cell sizes were five or fewer, Fisher's Exact tests were used. For continuous variables, two sample t-tests were used for parametric variables and Mann-Whitney U-tests were used for nonparametric variables. *P*-values were set at 0.05.

To assess the relationship between utilization of digital resources and whether the user reported that the digital platform improved their management of mental health, as well as the role of education and support as a mechanism, we used adjusted logistic regression. To specify the logistic regression models, we used the glm function in R and specified the family as binomial and the link as logit. For each model, we tested the assumptions (linearity, influential values, and multicollinearity) of logistic regression separately. To test linearity, we created scatter plots for continuous predictors and each outcome and examined the AIC for linear models compared to models with restricted cubic splines with 3, 4, and 5 knots. Results demonstrated that all linear models had the lowest AIC, and thus linear models were selected as the best fit for our outcomes. To test influential values, we examined Cook's distance values and found that there were no influential values in our data based on standardized residuals >3. To test multicollinearity, we examined the variance inflation factors (VIF) for each model and determined that there was no collinearity based on the observation that all VIF values were less than 5 (actual values were all <2). We used directed acyclic graphs (DAGs) to identify confounders and determine the covariates in each model (19).

**Table 1:** User characteristics and utilization by improvement in mental health management.

		Maven helped me manage anxiety/depression		
	Overall (*N* = 5659)	No (*N* = 4954, 87.5%)	Yes (*N* = 705, 12.5%)	*P* Value
**Demographics**				
Age [Mean (SD)]	32.7 (4.11)	32.7 (4.10)	32.7 (4.18)	0.67
Nulliparous	4,181 (73.9%)	3,683 (74.3%)	498 (70.6%)	0.036
Race/ethnicity				
Non-Hispanic White	2,361 (41.7%)	2,107 (42.5%)	254 (36.0%)	<0.001
Non-Hispanic Asian	1,030 (18.2%)	894 (18.0%)	136 (19.3%)	
Hispanic	464 (8.2%)	385 (7.8%)	79 (11.2%)	
Non-Hispanic Black	266 (4.7%)	217 (4.4%)	49 (7.0%)	
Non-Hispanic Multiracial/AI	129 (2.3%)	110 (2.2%)	19 (2.7%)	
Non-Hispanic Prefer not to say	1,409 (24.9%)	1,241 (25.1%)	168 (23.8%)	
**Risk**				
High SVI	168 (3.0%)	144 (2.9%)	24 (3.4%)	0.47
**Medical risk score**				
0	3,409 (60.2%)	3,007 (60.7%)	402 (57.0%)	0.01
1	1,378 (24.4%)	1,213 (24.5%)	165 (23.4%)	
2	564 (10.0%)	483 (9.7%)	81 (11.5%)	
3	219 (3.9%)	178 (3.6%)	41 (5.8%)	
4	66 (1.2%)	58 (1.2%)	8 (1.1%)	
5	18 (0.3%)	11 (0.2%)	7 (1.0%)	
6	5 (0.1%)	4 (0.1%)	1 (0.1%)	
Mental health conditions	1,880 (33.2%)	1,520 (30.7%)	360 (51.1%)	<0.001
Anxiety	1,320 (23.3%)	1,051 (21.2%)	269 (38.2%)	<0.001
Depression	724 (12.8%)	567 (11.4%)	157 (22.3%)	<0.001
Perinatal mood disorder	118 (2.1%)	75 (1.5%)	43 (6.1%)	<0.001
Pregnancy-related anxiety	682 (12.1%)	547 (11.0%)	135 (19.1%)	<0.001
**Support Interests**				
Emotional health	4,022 (71.1%)	3,435 (69.3%)	587 (83.3%)	<0.001
**Utilization**				
Trimester Enrolled				
1st Trimester	1,552 (27.4%)	1,296 (26.2%)	256 (36.3%)	<0.001
2nd Trimester	2,449 (43.3%)	2,127 (42.9%)	322 (45.7%)	
3rd Trimester	1,658 (29.3%)	1,531 (30.9%)	127 (18.0%)	
Educational Content				
Articles read^4^				
Q1: [0,8]	1,456 (25.7%)	1,344 (27.1%)	112 (15.9%)	<0.001
Q2: (8,22]	1,376 (24.3%)	1,230 (24.8%)	146 (20.7%)	
Q3: (22,50]	1,446 (25.6%)	1,241 (25.1%)	205 (29.1%)	
Q4: (50,423]	1,381 (24.4%)	1,139 (23.0%)	242 (34.3%)	
Read at least one mental health article	521 (9.2%)	400 (8.1%)	121 (17.2%)	<0.001
Class recordings watched^4^				
Q1: 0	2,827 (50.0%)	2,540 (51.3%)	287 (40.7%)	<0.001
Q2: (0,1]	809 (14.3%)	694 (14.0%)	115 (16.3%)	
Q3: (1,3]	772 (13.6%)	652 (13.2%)	120 (17.0%)	
Q4: (3,103]	1,251 (22.1%)	1,068 (21.6%)	183 (26.0%)	
Virtual classes attended^4^				0.17
Q1/Q2: 0	3,099 (54.8%)	2,722 (54.9%)	377 (53.5%)	
Q3: (0,2]	1,322 (23.4%)	1,167 (23.6%)	155 (22.0%)	
Q4: (2,22]	1,238 (21.9%)	1,065 (21.5%)	173 (24.5%)	
Care Coordination				
Messages sent to Care Advocate^4^				<0.001
Q1: 0	2,265 (40.0%)	2,083 (42.0%)	182 (25.8%)	
Q2: (0,1]	770 (13.6%)	682 (13.8%)	88 (12.5%)	
Q3: (1,5]	1,363 (24.1%)	1,179 (23.8%)	184 (26.1%)	
Q4: (5,96]	1,261 (22.3%)	1,010 (20.4%)	251 (35.6%)	
At least one CA appointment	3,864 (68.3%)	3,310 (66.8%)	554 (78.6%)	<0.001
Provider Services				
Messages sent to providers^4^				<0.001
1/Q2: 0	3,522 (62.2%)	3,231 (65.2%)	291 (41.3%)	
Q3: (0,2]	871 (15.4%)	748 (15.1%)	123 (17.4%)	
Q4: (2,376]	1,266 (22.4%)	975 (19.7%)	291 (41.3%)	
Any message sent to a mental health provider	251 (4.4%)	122 (2.5%)	129 (18.3%)	<0.001
Total virtual provider appointments attended^4^				<0.001
Q1: 0	2,655 (46.9%)	2,472 (49.9%)	183 (26.0%)	
Q2: (0,1]	947 (16.7%)	842 (17.0%)	105 (14.9%)	
Q3: (1,3]	990 (17.5%)	857 (17.3%)	133 (18.9%)	
Q4: (3,117]	1,067 (18.9%)	783 (15.8%)	284 (40.3%)	
At least one appointment with:				
Any provider	3,004 (53.1%)	2,482 (50.1%)	522 (74.0%)	<0.001
OB/GYN providers	1,114 (19.7%)	892 (18.0%)	222 (31.5%)	<0.001
Mental health providers	513 (9.1%)	247 (5.0%)	266 (37.7%)	<0.001
Midwifes	276 (4.9%)	223 (4.5%)	53 (7.5%)	<0.001
Doulas	1,291 (22.8%)	1,042 (21.0%)	249 (35.3%)	<0.001
**Education and Support**				
Maven helped me understand warning signs during pregnancy^2^	3,211 (56.7%)	2,695 (54.4%)	516 (73.2%)	<0.001
Maven helped me learn medically accurate information about pregnancy and/or complications	2,774 (49.0%)	2,375 (47.9%)	399 (56.6%)	<0.001
On a scale of 1–5, how would you rate the support you received from Maven on your pregnancy journey?				<0.001
<3	1,471 (26.0%)	1,413 (28.5%)	58 (8.2%)	
4	2,319 (41.0%)	2,037 (41.1%)	282 (40.0%)	
5	1,869 (33.0%)	1,504 (30.4%)	365 (51.8%)	

*Aim 1.* To assess *if* using digital services and resources has an effect on perceived improvements in management of depression and anxiety during pregnancy, we assessed each utilization type in a separate logistic regression model. Adjusted models controlled for age, race/ethnicity, trimester enrolled, and high SVI. Models were stratified by the presence of mental health conditions.

*Aim 2a.* To examine *how* using digital services and resources may influence management of mental health through the pathways of education and support as the drivers of improvement, we first examined the relationship between utilization and education and support. We assessed each utilization type in a separate logistic regression for each indicator of education or support including: (1) understanding warning signs, (2) learning medically accurate information about pregnancy, and (3) receiving excellent support through the digital platform. For support through the platform, multinomial logistic regression was used. Models were adjusted for SVI and parity. These models were not stratified, as we did not anticipate that the effect of utilization on education or perceived support would differ based on existing mental health conditions.

*Aim 2b.* To assess the effect of education and support on reported improvement in mental health management, we used adjusted logistic regression. Models were stratified by existing mental health conditions, and adjusted for SVI, parity, and trimester enrolled.

All analyses were conducted in R v.3.6.3 (20).

## Results

### Sample description

**Figure 2:**
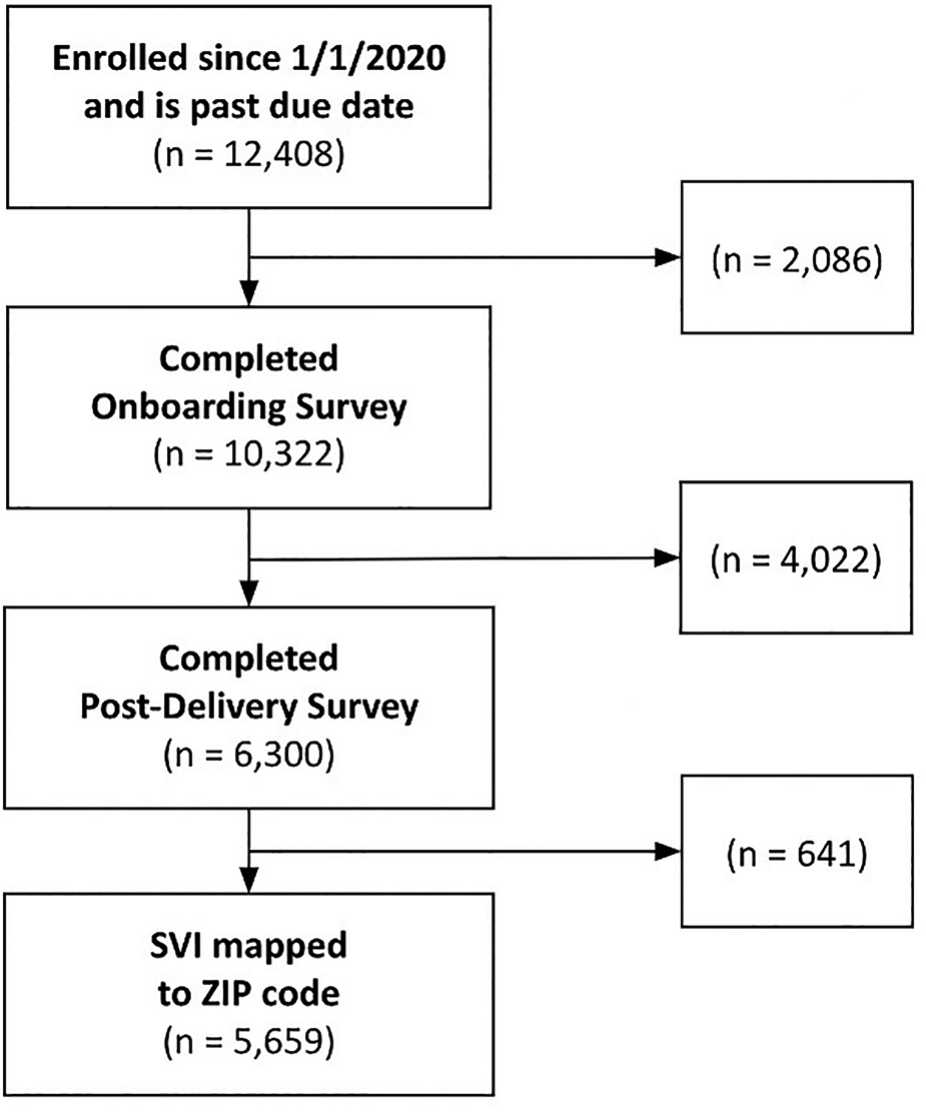
Flow diagram.

The final study population included 5,659 pregnant people who met all inclusion criteria (Figure 2). The average age of the sample was 32.7 (SD = 4.11) years, and 4,181 users (73.9%) were nulliparous (Table 1). The majority of the users (2361; 41.7%) identified as non-Hispanic White, 1,030 users (18.2%) as Asian, 464 (8.2%) as Hispanic, 266 (4.7%) as Black, and 129 (2.3%) as multiracial, American Indian, or other. In the study population 24.9% of people did not disclose their racial and ethnic identity. The majority of users reported no existing medical conditions (3409; 60.2%), and 168 (3.0%) of the sample had a high SVI. Of the total sample, 1,880 (33.2%) reported at least one mental health condition: 1,320 (23.3%) had anxiety, 724 (12.8%) had depression, 118 (2.1%) had perinatal mood disorder, and 682 (12.1%) had pregnancy-related anxiety (Table 1).

In the study population, 705 users (12.5%) reported that Maven helped them manage anxiety or depression during pregnancy. The majority of users (56.7%) reported that Maven helped them understand warning signs, and 49.0% said Maven helped them learn medically accurate information. When asked about the support they received, 41.0% (2319/5659) rated it as 4 (good) and 33.0% (1869/5659) rated it as 5 (excellent) (Table 1).

The population of users that reported improved mental health management in their pregnancy were more likely to identify as non-white (*p* < 0.001), more likely to report at least one medical condition (*p* = 0.01), report a mental health condition (*p* < 0.001), and report wanting support for emotional health (*p* < 0.001). They were also significantly more likely to use all digital resources (*p* < 0.001) except for virtual class attendance, which was not significant (*p* = 0.17), and to have enrolled in the platform earlier in their pregnancy (*p* < 0.001). This population was also more likely to report that the platform: helped them understand warning signs (*p* < 0.001), helped them learn medically accurate information about pregnancy and/or complications (*p* < 0.001), and provided them with excellent support (*p* < 0.001) (Table 1).

### Effect of digital service utilization on perceived help with managing anxiety and depression

**Figure 3:**
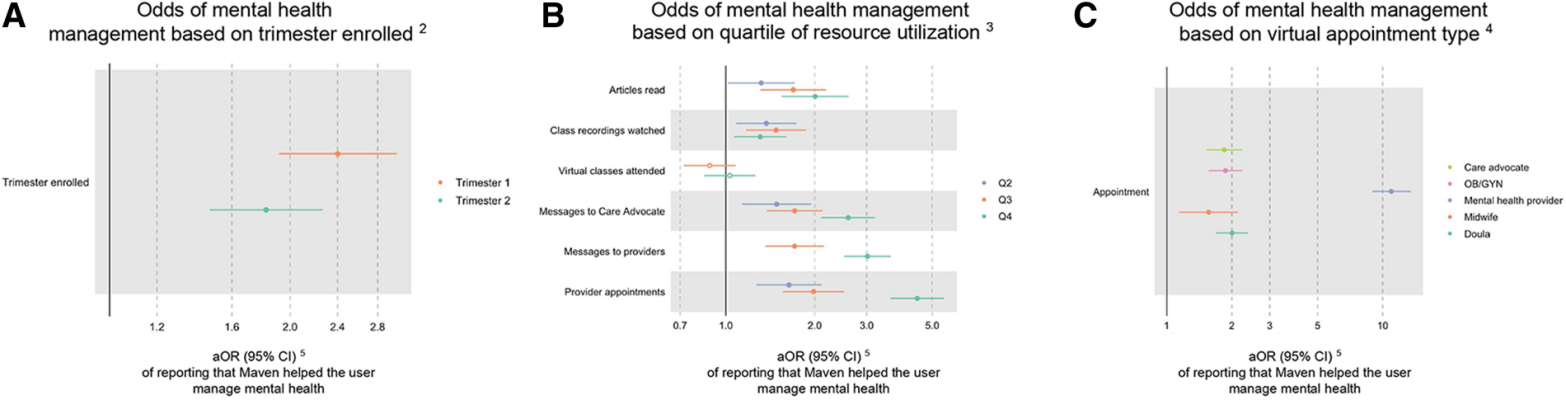
Effect of utilization on mental health management in total sample^1^. (**A**) Odds of mental health management based on trimester enrolled^2^. (**B**) Odds of mental health management based on quartile of resource utilization^3^. (**C**) Odds of mental health management based on virtual appointment type^4^. ^1^Filled data points represent statistically significant findings (*p* < 0.05), and unfilled data points represent findings that are not statistically significant. ^2^Compared to Trimester 3 enrollment. ^3^Compared to Quartile 1 utilization. ^4^Compared to users who did not have an appointment with each provider type. ^5^Adjusted odds ratio (95% confidence interval).

In adjusted models, earlier enrollment in the digital platform was associated with higher odds of perceived improved mental health management in pregnancy (first trimester enrollment: aOR 2.41 (95% CI 1.93, 3.03); second trimester enrollment: aOR 1.83 (95% CI 1.48, 2.28)) when compared to third trimester enrollment (Table 2; Figure 3A). More articles read and class recordings viewed were both associated with reported improved management of mental health (e.g., articles read: Q2 aOR 1.31 (95% CI 1.01, 1.70), Q3 aOR 1.68 (95% CI 1.30, 2.17), Q4 1.99 (95% CI 1.54, 2.59)) (Table 2; Figure 3B). Care coordination through messages or appointments with a care advocate were also associated with an increased odds of reporting the platform helped with mental health management (e.g., care advocate messages sent: Q2 aOR 1.48 (95% CI 1.12, 1.93), Q3 aOR 1.70 (95% CI 1.36, 2.12), Q4 2.58 (95% CI 2.10, 3.18)) (Table 2; Figure 3B). Utilization of mental health providers was highly associated with increased odds of reporting improved mental health management (one or more appointment with a mental health provider aOR of 11.0 (95% CI 8.95, 13.4); one or more messages to a mental health provider aOR of 8.45 (95% CI 6.47, 11.0)) (Table 2; Figure 3C). Overall, greater utilization of digital resources and services was associated with reported improvements in mental health management in pregnancy in both the cohort with existing mental health conditions and the cohort without these conditions (Table 2).

**Table 2:** Effect of utilization on mental health management by presence of a mental health condition.

	Total Sample (*N* = 5659)		Existing Depression, Anxiety, or Pregnancy Related Anxiety (*N* = 1880)		No Existing Depression, Anxiety, or Pregnancy Related Anxiety (*N* = 3779)	
	OR (95% CI)	aOR (95% CI)^a^	OR (95% CI)	aOR (95% CI)^a^	OR (95% CI)	aOR (95% CI)^a^
**Trimester Enrolled**						
First trimester	2.38 (1.91, 2.99)	2.41 (1.93, 3.03)	2.07 (1.50, 2.88)	2.12 (1.53, 2.97)	2.24 (1.63, 3.09)	2.25 (1.64, 3.11)
Second trimester	1.82 (1.47, 2.27)	1.83 (1.48, 2.28)	1.58 (1.15, 2.20)	1.6 (1.16, 2.23)	1.96 (1.47, 2.65)	1.96 (1.46, 2.65)
Third trimester	Ref	Ref	Ref	Ref	Ref	Ref
**Educational Content**						
Articles read						
Q1	Ref	Ref	Ref	Ref	Ref	Ref
Q2	1.42 (1.10, 1.85)	1.31 (1.01, 1.70)	1.29 (0.90, 1.86)	1.18 (0.82, 1.71)	1.56 (1.07, 2.29)	1.46 (1.00, 2.15)
Q3	1.98 (1.56, 2.53)	1.68 (1.30, 2.17)	1.83 (1.30, 2.60)	1.57 (1.09, 2.28)	2.28 (1.62, 3.26)	1.97 (1.37, 2.85)
Q4	2.55 (2.02, 3.24)	1.99 (1.54, 2.59)	2.25 (1.62, 3.16)	1.8 (1.25, 2.62)	2.91 (2.07, 4.14)	2.39 (1.65, 3.50)
Mental health articles read						
Any	2.36 (1.89, 2.93)	1.97 (1.57, 2.47)	2.54 (1.84, 3.47)	2.26 (1.62, 3.13)	2.05 (1.47, 2.81)	1.69 (1.20, 2.35)
Class recordings watched						
Q1	Ref	Ref	Ref	Ref	Ref	Ref
Q2	1.47 (1.16, 1.84)	1.37 (1.08, 1.72)	1.29 (0.92, 1.80)	1.22 (0.87, 1.71)	1.65 (1.18, 2.27)	1.53 (1.09, 2.13)
Q3	1.63 (1.29, 2.04)	1.47 (1.16, 1.86)	1.64 (1.17, 2.30)	1.56 (1.10, 2.19)	1.73 (1.25, 2.38)	1.54 (1.10, 2.14)
Q4	1.52 (1.24, 1.85)	1.31 (1.06, 1.60)	1.35 (1.00, 1.81)	1.17 (0.86, 1.59)	1.77 (1.34, 2.33)	1.53 (1.15, 2.03)
Virtual classes attended						
Q1 and Q2	Ref	Ref	Ref	Ref	Ref	Ref
Q3	0.96 (0.78, 1.17)	0.88 (0.72, 1.08)	0.85 (0.64, 1.14)	0.81 (0.60, 1.08)	1.05 (0.79, 1.38)	0.96 (0.72, 1.26)
Q4	1.17 (0.96, 1.42)	1.03 (0.84, 1.25)	1.17 (0.87, 1.56)	1.06 (0.78, 1.42)	1.26 (0.96, 1.63)	1.1 (0.83, 1.44)
**Care Coordination**						
Messages sent to Care Advocate						
Q1	Ref	Ref	Ref	Ref	Ref	Ref
Q2	1.48 (1.12, 1.93)	1.48 (1.12, 1.93)	1.56 (1.04, 2.30)	1.52 (1.01, 2.25)	1.43 (0.97, 2.06)	1.44 (0.98, 2.08)
Q3	1.79 (1.44, 2.22)	1.70 (1.36, 2.12)	1.94 (1.41, 2.67)	1.84 (1.33, 2.54)	1.69 (1.25, 2.29)	1.63 (1.20, 2.20)
Q4	2.84 (2.32, 3.50)	2.58 (2.10, 3.18)	2.95 (2.19, 3.98)	2.71 (2.00, 3.68)	2.66 (2.00, 3.55)	2.38 (1.78, 3.20)
At least one appointment with a CA	1.82 (1.51, 2.21)	1.84 (1.52, 2.23)	2.24 (1.70, 2.97)	2.3 (1.74, 3.08)	1.58 (1.23, 2.07)	1.57 (1.21, 2.05)
**Provider Services**						
Messages sent to care providers						
Q1 and Q2	Ref	Ref	Ref	Ref	Ref	Ref
Q3	1.83 (1.45, 2.28)	1.71 (1.36, 2.14)	1.69 (1.22, 2.34)	1.61 (1.15, 2.23)	1.84 (1.33, 2.52)	1.70 (1.23, 2.33)
Q4	3.31 (2.77, 3.96)	3.02 (2.52, 3.62)	3.28 (2.52, 4.26)	3.07 (2.34, 4.01)	3.25 (2.54, 4.16)	2.94 (2.28, 3.79)
Messages sent to mental health providers						
Any	8.87 (6.83, 11.5)	8.45 (6.47, 11.0)	7.11 (4.94, 10.3)	6.79 (4.69, 9.88)	9.06 (6.16, 13.3)	9.17 (6.17, 13.6)
Total virtual provider appointments attended						
Q1	Ref	Ref	Ref	Ref	Ref	Ref
Q2	1.68 (1.31, 2.16)	1.64 (1.27, 2.10)	1.38 (0.92, 2.02)	1.38 (0.92, 2.03)	1.96 (1.40, 2.73)	1.86 (1.33, 2.60)
Q3	2.10 (1.65, 2.65)	1.98 (1.56, 2.51)	2.26 (1.61, 3.18)	2.21 (1.56, 3.12)	1.90 (1.35, 2.65)	1.77 (1.25, 2.47)
Q4	4.90 (4.01, 6.02)	4.44 (3.61, 5.48)	4.99 (3.72, 6.74)	4.69 (3.46, 6.39)	4.51 (3.40, 6.00)	4.02 (3.01, 5.39)
Attended at least one appointment, by type						
OB/GYN providers	2.09 (1.76, 2.49)	1.87 (1.56, 2.24)	2.21 (1.71, 2.85)	2.01 (1.54, 2.60)	1.92 (1.49, 2.45)	1.69 (1.31, 2.18)
Mental health providers	11.5 (9.47, 14.1)	11.0 (8.95, 13.4)	9.89 (7.50, 13.1)	9.57 (7.22, 12.7)	10.7 (7.99, 14.4)	10.5 (7.80, 14.2)
Midwives	1.72 (1.25, 2.33)	1.56 (1.13, 2.12)	1.44 (0.87, 2.30)	1.27 (0.76, 2.05)	2.03 (1.33, 3.01)	1.86 (1.21, 2.77)
Doulas	2.05 (1.73, 2.43)	2.01 (1.69, 2.38)	2.28 (1.77, 2.92)	2.28 (1.76, 2.93)	1.9 (1.50, 2.41)	1.82 (1.43, 2.31)

^a^ Models were adjusted for age, race/ ethnicity, trimester enrolled, SVI.

### Effect of digital service utilization on education and support

**Figure 4:**
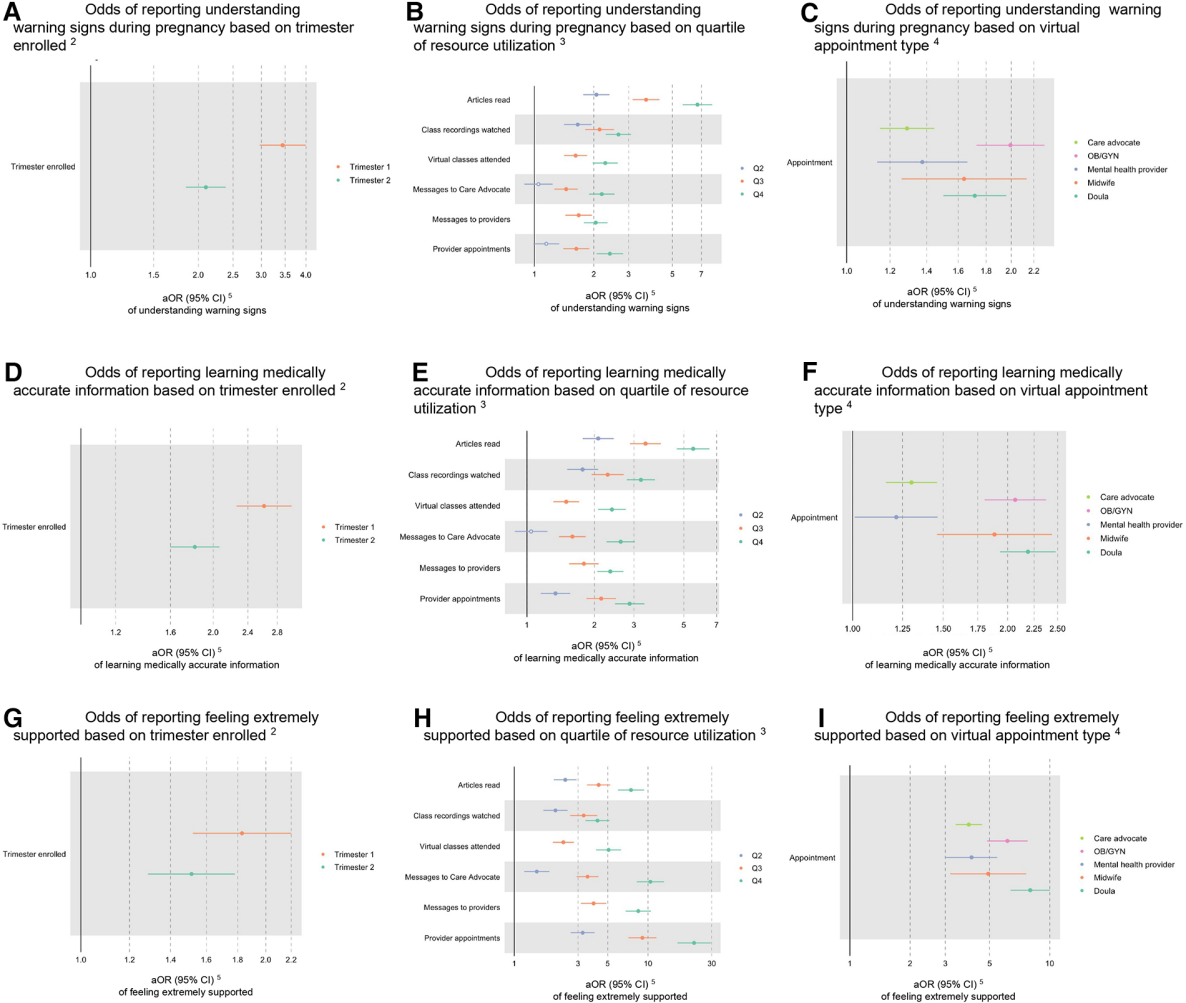
Effect of utilization on understanding warning signs in pregnancy, learning medically accurate information about pregnancy, and feeling supported in total sample^1^. (**A**) Odds of reporting understanding warning signs during pregnancy based on trimester enrolled^2^. (**B**) Odds of reporting understanding warning signs during pregnancy based on quartile of resource utilization^3^. (**C**) Odds of reporting understanding warning signs during pregnancy based on virtual appointment type^4^. (**D**) Odds of reporting learning medically accurate information based on trimester enrolled^2^. (**E**) Odds of reporting learning medically accurate information based on quartile of resource utilization^3^. (**F**) Odds of reporting learning medically accurate information based on virtual appointment type^4^. (**G**) Odds of reporting feeling extremely supported based on trimester enrolled^2^. (**H**) Odds of reporting feeling extremely supported based on quartile of resource utilization^3^. (**I**) Odds of reporting feeling extremely supported based on virtual appointment type^4^. ^1^Filled data points represent statistically significant findings (*p* < 0.05), and unfilled data points represent findings that are not statistically significant. ^2^Compared to Trimester 3 enrollment. ^3^Compared to Quartile 1 utilization. ^4^Compared to users who did not have an appointment with each provider type. ^5^Adjusted odds ratio (95% confidence interval).

Earlier enrollment to the digital platform and greater use of the platform across all categories of use were associated with higher likelihood of helping users gain knowledge around pregnancy in a dose response pattern. In adjusted models, first trimester enrollment (aOR 3.44; 95% CI 2.97–3.99) and second trimester enrollment (aOR 2.10; 95% CI 1.85–2.39) were associated with increased understanding of warning signs compared to third trimester enrollment (Table 3; Figure 4A). This pattern held in reports of learning medically accurate information (Table 3; Figure 4D). Articles read had the greatest effect on reporting increased education for both measures (e.g., warning signs: Q2 aOR 2.06; 95% CI: 1.77–2.40; Q3 aOR 3.68; 95% CI: 3.15–4.30; Q4 aOR 6.69; 95% CI: 5.64–7.96) (Table 3; Figure 4). Reading at least one mental health article specifically had an aOR of 2.87 (95% CI 2.32–3.58) for helping users understand warning signs in pregnancy and an aOR of 2.11 (95% CI 1.74–2.56) for helping users learn medically accurate information about pregnancy (Table 3). A dose response was observed for messages to a care advocate or provider and for appointments with providers (Table 3; Figure 4). At least one appointment with an OB/GYN, mental health provider, midwife, or doula each had an impact on the educational outcomes of interest, with an OB/GYN appointment having the largest effect on understanding warning signs (aOR 1.99; 95% CI 1.73–2.30) (Table 3; Figure 4C) and a doula appointment having the largest effect on learning medically accurate information (aOR 2.19; 95% CI 1.92–2.50) (Table 3; Figure 4F).

**Table 3:** effect of utilization on understanding warning signs in pregnancy, learning medically accurate information about pregnancy, and feeling supported.

	Maven helped me understand warning signs during pregnancy		Maven helped me learn medically accurate information about pregnancy and/or complications		On a scale of 1-5, how would you rate the support you received from Maven on your pregnancy journey?	
	OR (95% CI)	aOR (95% CI)^a^	OR (95% CI)	aOR (95% CI)^a^	OR (95% CI)	aOR (95% CI)^a^
**Trimester Enrolled**						
First trimester	3.53 (3.05, 4.09)	3.44 (2.97, 3.99)	2.69 (2.34, 3.11)	2.61 (2.26, 3.02)	Very good: 1.71 (1.44, 2.04) Excellent: 1.9 (1.58, 2.28)	Very good: 1.67 (1.40, 1.99) Excellent: 1.83 (1.52, 2.20)
Second trimester	2.15 (1.90, 2.44)	2.10 (1.85, 2.39)	1.87 (1.65, 2.13)	1.82 (1.60, 2.07)	Very good: 1.46 (1.25, 1.70) Excellent: 1.56 (1.33, 1.84)	Very good: 1.43 (1.22, 1.66) Excellent: 1.51 (1.29, 1.78)
Third trimester	Ref	Ref	Ref	Ref	Ref	Ref
**Educational Content**						
Articles read						
Q1	Ref	Ref	Ref	Ref	Ref	Ref
Q2	2.07 (1.78, 2.41)	2.06 (1.77, 2.40)	2.16 (1.84, 2.53)	2.08 (1.77, 2.44)	Very good: 2.36 (1.98, 2.81) Excellent: 2.47 (2.03, 3.00)	Very good: 2.32 (1.94, 2.77) Excellent: 2.41 (1.98, 2.93)
Q3	3.74 (3.21, 4.37)	3.68 (3.15, 4.30)	3.60 (3.08, 4.21)	3.38 (2.88, 3.96)	Very good: 3.30 (2.74, 3.97) Excellent: 4.47 (3.67, 5.46)	Very good: 3.22 (2.67, 3.88) Excellent: 4.27 (3.50, 5.22)
Q4	6.97 (5.90, 8.25)	6.69 (5.64, 7.96)	6.13 (5.21, 7.22)	5.50 (4.66, 6.51)	Very good: 5.51 (4.47, 6.79) Excellent: 8.02 (6.44, 10.0)	Very good: 5.30 (4.28, 6.56) Excellent: 7.46 (5.96, 9.34)
Prenatal mental health articles	2.97 (2.41, 3.70)	2.87 (2.32, 3.58)	2.21 (1.83, 2.67)	2.11 (1.74, 2.56)	Very good: 1.94 (1.48, 2.56) Excellent: 2.84 (2.16, 3.73)	Very good: 1.89 (1.43, 2.49) Excellent: 2.71 (2.06, 3.57)
Class recordings watched						
Q1	Ref	Ref	Ref	Ref	Ref	Ref
Q2	1.72 (1.46, 2.01)	1.66 (1.41, 1.95)	1.86 (1.59, 2.18)	1.77 (1.51, 2.08)	Very good: 1.66 (1.36, 2.03) Excellent: 2.11 (1.72, 2.59)	Very good: 1.63 (1.33, 1.98) Excellent: 2.03 (1.65, 2.50)
Q3	2.26 (1.91, 2.67)	2.14 (1.81, 2.53)	2.47 (2.10, 2.91)	2.29 (1.94, 2.70)	Very good: 2.72 (2.17, 3.41) Excellent: 3.51 (2.78, 4.43)	Very good: 2.62 (2.09, 3.29) Excellent: 3.31 (2.62, 4.18)
Q4	2.91 (2.52, 3.36)	2.66 (2.30, 3.09)	3.61 (3.14, 4.17)	3.22 (2.79, 3.72)	Very good: 3.43 (2.81, 4.17) Excellent: 4.57 (3.74, 5.60)	Very good: 3.25 (2.66, 3.97) Excellent: 4.19 (3.41, 5.15)
Virtual classes attended						
Q1 and Q2	Ref	Ref	Ref	Ref	Ref	Ref
Q3	1.72 (1.51, 1.96)	1.62 (1.41, 1.85)	1.64 (1.44, 1.87)	1.50 (1.31, 1.71)	Very good: 2.11 (1.78, 2.49) Excellent: 2.44 (2.05, 2.91)	Very good: 2.05 (1.72, 2.43) Excellent: 2.33 (1.95, 2.79)
Q4	2.52 (2.19, 2.91)	2.28 (1.97, 2.65)	2.77 (2.42, 3.19)	2.39 (2.08, 2.76)	Very good: 3.68 (2.99, 4.52) Excellent: 5.48 (4.45, 6.76)	Very good: 3.50 (2.83, 4.32) Excellent: 5.07 (4.09, 6.29)
**Care Coordination**						
Messages sent to Care Advocate						
Q1	Ref	Ref	Ref	Ref	Ref	Ref
Q2	1.03 (0.88, 1.22)	1.05 (0.89, 1.24)	1.04 (0.88, 1.23)	1.04 (0.88, 1.23)	Very good: 1.32 (1.09, 1.60) Excellent: 1.47 (1.18, 1.82)	Very good: 1.32 (1.09, 1.60) Excellent: 1.47 (1.18, 1.83)
Q3	1.46 (1.27, 1.67)	1.45 (1.26, 1.66)	1.62 (1.42, 1.86)	1.59 (1.39, 1.83)	Very good: 2.29 (1.92, 2.72) Excellent: 3.58 (2.97, 4.31)	Very good: 2.25 (1.89, 2.68) Excellent: 3.53 (2.93, 4.26)
Q4	2.31 (2.00, 2.68)	2.19 (1.89, 2.54)	2.81 (2.44, 3.25)	2.62 (2.27, 3.03)	Very good: 4.62 (3.67, 5.81) Excellent: 11.0 (8.71, 13.9)	Very good: 4.42 (3.51, 5.57) Excellent: 10.40 (8.24, 13.2)
At least one CA appointment	1.28 (1.14, 1.43)	1.29 (1.15, 1.44)	1.29 (1.16, 1.45)	1.30 (1.16, 1.46)	Very good: 2.64 (2.30, 3.03) Excellent: 3.87 (3.33, 4.50)	Very good: 2.66 (2.32, 3.05) Excellent: 3.93 (3.38, 4.58)
**Provider Services**						
Messages sent to care providers						
Q1 and Q2	Ref	Ref	Ref	Ref	Ref	Ref
Q3	1.71 (1.47, 1.99)	1.67 (1.44, 1.96)	1.84 (1.58, 2.14)	1.79 (1.54, 2.09)	Very good: 2.36 (1.91, 2.92) Excellent: 3.99 (3.21, 4.96)	Very good: 2.33 (1.88, 2.88) Excellent: 3.91 (3.15, 4.87)
Q4	2.10 (1.83, 2.40)	2.04 (1.78, 2.34)	2.42 (2.12, 2.76)	2.35 (2.06, 2.69)	Very good: 3.37 (2.71, 4.18) Excellent: 8.64 (6.96, 10.7)	Very good: 3.31 (2.67, 4.12) Excellent: 8.45 (6.81, 10.5)
Messages sent to mental health providers	1.38 (1.07, 1.81)	1.44 (1.11, 1.89)	1.20 (0.93, 1.55)	1.27 (0.98, 1.65)	Very good: 1.96 (1.31, 2.94) Excellent: 3.14 (2.11, 4.66)	Very good: 2.06 (1.38, 3.10) Excellent: 3.36 (2.26, 5.01)
Total virtual provider appointments attended						
Q1	Ref	Ref	Ref	Ref	Ref	Ref
Q2	1.14 (0.99, 1.33)	1.15 (0.99, 1.33)	1.33 (1.14, 1.54)	1.34 (1.15, 1.56)	Very good: 2.19 (1.82, 2.64) Excellent: 3.2 (2.61, 3.93)	Very good: 2.22 (1.84, 2.67) Excellent: 3.25 (2.64, 3.99)
Q3	1.70 (1.46, 1.97)	1.63 (1.40, 1.90)	2.24 (1.93, 2.61)	2.14 (1.84, 2.49)	Very good: 4.62 (3.67, 5.82) Excellent: 9.4 (7.39, 11.9)	Very good: 4.51 (3.58, 5.68) Excellent: 9.08 (7.14, 11.5)
Q4	2.50 (2.15, 2.92)	2.41 (2.07, 2.82)	2.99 (2.58, 3.47)	2.87 (2.47, 3.34)	Very good: 6.98 (5.24, 9.29) Excellent: 22.8 (17.1, 30.3)	Very good: 6.82 (5.12, 9.09) Excellent: 22.10 (16.6, 29.5)
At least one appointment with:						
OB/GYN providers	2.07 (1.80, 2.39)	1.99 (1.73, 2.30)	2.17 (1.90, 2.49)	2.07 (1.80, 2.37)	Very good: 3.64 (2.88, 4.59) Excellent: 6.40 (5.08, 8.08)	Very good: 3.53 (2.79, 4.46) Excellent: 6.14 (4.86, 7.75)
Mental health providers	1.32 (1.09, 1.59)	1.38 (1.14, 1.66)	1.15 (0.96, 1.38)	1.22 (1.01, 1.46)	Very good: 2.34 (1.73, 3.15) Excellent: 3.80 (2.83, 5.10)	Very good: 2.45 (1.82, 3.31) Excellent: 4.06 (3.02, 5.46)
Midwife	1.76 (1.36, 2.29)	1.64 (1.26, 2.14)	2.05 (1.59, 2.65)	1.89 (1.46, 2.44)	Very good: 2.75 (1.75, 4.31) Excellent: 5.30 (3.43, 8.20)	Very good: 2.60 (1.66, 4.08) Excellent: 4.92 (3.18, 7.62)
Doula	1.84 (1.61, 2.10)	1.72 (1.50, 1.96)	2.36 (2.08, 2.69)	2.19 (1.92, 2.50)	Very good: 3.85 (3.07, 4.84) Excellent: 8.44 (6.73, 10.6)	Very good: 3.69 (2.94, 4.65) Excellent: 7.98 (6.36, 10.0)

Note: *n* = 5659.

^a^ Models were adjusted for SVI, parity.

Utilization categories showed a similar relationship with perceived support from the platform, such that higher utilization was more strongly associated with very good and excellent support in a dose response manner. The highest quartile of use for provider appointments had an aOR of 6.82 (95% CI 5.12–9.09) for very good support and an aOR of 22.10 (95% CI 16.6–29.5) for excellent support (Table 3; Figure 4H).

### Effect of education and support on perceived help with management of anxiety and depression

**Figure 5:**
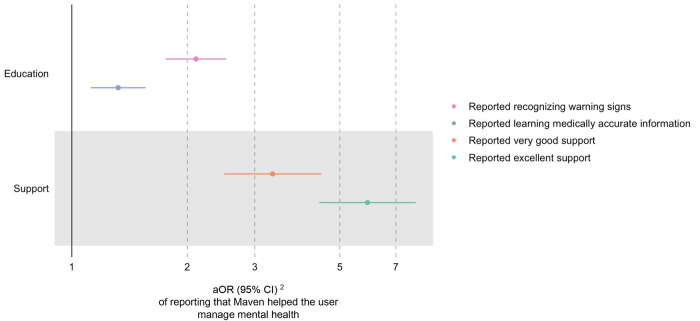
Effect of education and support on mental health management in total sample^1^. ^1^Filled data points represent statistically significant findings (*p* < 0.05), and unfilled data points represent findings that are not statistically significant. ^2^Adjusted odds ratio (95% confidence interval).

Users were more likely to report better mental health management in pregnancy if they reported that the digital platform helped them recognize warning signs (aOR 2.11; 95% CI 1.76, 2.53), learn medically accurate information (aOR 1.32; 95% CI 1.12, 1.56), or rated support from the platform as very good (aOR 3.34; 95% CI 2.51, 4.51) or excellent (aOR 5.90; 95% CI 4.45, 7.95) (Table 4; Figure 5).

**Table 4:** Effect of education and support on mental health management.

	Total Sample (*N* = 5659)		Existing Depression, Anxiety, and Pregnancy Related Anxiety (*N* = 1880)		No Existing Depression, Anxiety, and Pregnancy Related Anxiety (*N* = 3779)	
	OR (95% CI)	aOR (95% CI)^a^	OR (95% CI)	aOR (95% CI)^a^	OR (95% CI)	aOR (95% CI)^a^
**Education**						
Maven helped me understand warning signs during pregnancy	2.29 (1.92, 2.73)	2.11 (1.76, 2.53)	2.76 (2.12, 3.62)	2.66 (2.03, 3.51)	1.88 (1.48, 2.39)	1.71 (1.34, 2.19)
Maven helped me learn medically accurate information about pregnancy and/or complications	1.42 (1.21, 1.66)	1.32 (1.12, 1.56)	1.27 (1.01, 1.60)	1.24 (0.98, 1.58)	1.52 (1.21, 1.90)	1.39 (1.11, 1.76)
**Maven Support**						
Very Good	3.37 (2.54, 4.55)	3.34 (2.51, 4.51)	4.36 (2.85, 6.92)	4.42 (2.88, 7.04)	2.74 (1.88, 4.11)	2.67 (1.83, 4.02)
Excellent	5.91 (4.48, 7.95)	5.90 (4.45, 7.95)	7.40 (4.87, 11.7)	7.67 (5.02, 12.2)	4.95 (3.43, 7.37)	4.84 (3.34, 7.22)

^a^ Models were adjusted for SVI, parity, trimester enrolled.

## Discussion

Educational content, care coordination, and provider services all had a positive effect on users' report that a digital platform helped them manage anxiety or depression during their pregnancy. A dose response was observed for reading articles, messaging care advocates or providers, and having appointments with providers. The positive effects of utilization on perceived management of mental health were observed both in users with and without diagnosed existing mental health conditions. Results show that the relationship between digital service utilization and reported improved mental health management are at least in part through pathways of education about pregnancy and support during this critical time period.

The results of this study support the existing body of literature showing that digital platforms are a valuable option for filling gaps in mental healthcare (10–12). Previous research has found a benefit in clinical outcomes and patient-reported well-being and satisfaction for people who leveraged telehealth services (21). Remotely delivered interventions, such as guided meditation or engaging in behavioral health care, have been shown to help reduce anxiety and improve overall mental health during pregnancy (13, 22, 23).

While mental health specific digital platforms have been shown to drive improved mental health outcomes for their users, their benefits inherently elude people who do not specifically seek out mental health support. In our study, users who did not have an existing mental health condition, and therefore may not have sought out a mental health app, also reported benefits on management of mental health both from the platform's general and mental health specific content and support. Due in part to mental health care shortages, mental health conditions are under-diagnosed, and many people without a diagnosed mental health condition still grapple with symptoms of anxiety and depression (24). Digital platforms that offer comprehensive care may have the opportunity to seamlessly guide users to resources that have benefits for depression and anxiety, even for people who do not specifically recognize the need for or seek out these services.

In addition to benefiting from mental health specific resources on the comprehensive platform, our results also indicate that increasing knowledge around pregnancy can help pregnant people manage anxiety or depression. Even asynchronous education through reading articles and viewing videos had a positive influence on mental health management. This is consistent with previous findings that demonstrated that receiving information around complications in pregnancy reduces maternal anxiety (25). Mental health management has traditionally been approached through mental health specific resources, but our results demonstrate that mental health management during pregnancy can be improved through additional education about pregnancy. This finding is particularly important within the context of the shortage of mental health personnel in the United States, as digital solutions offer a cost-effective strategy to improve outcomes and can help to redistribute mental health personnel resources to those who will benefit the most.

Support is another viable pathway for improving mental health management during pregnancy. Our results add evidence to a body of literature that has found that social support can play a role in mental health management (26, 27). However, the concept of support *via* digital solutions is relatively new and unexplored. One narrative review posits that social support *via* technology can facilitate the digital therapeutic alliance between provider and patient (28, 29). In our study, perceived support was associated with improved management of mental health for both pregnant people with and without a mental health condition, but the effect was greater for those with an existing mental health condition.

### Strengths

This analysis has several strengths. First, we assessed a national sample of commercially insured pregnant people that is not limited to a single health care system. Second, our unique data set included users' perceptions of the influence of digital services on support, education, and mental health outcomes, allowing us to better understand the mechanisms through which digital services may improve mental health management. We also capture anxiety as it relates to pregnancy, a concept that is typically not screened for in routine prenatal care. Lastly, we leveraged product utilization data that are tracked internally, so they are not subject to recall bias.

### Limitations

Except for utilization data, all data were self-reported by users, which may cause misreporting of medical conditions and a lack of standardization in the mental health outcome of interest. The assessment of medical conditions as a score does not account for the difference in severity of condition, or conditions relative to each other. However, self-report data also provides a patient-centered perspective on the value of digital solutions. Our outcome inherently included a user's perception of whether the platform helped them manage their anxiety or depression. It could also reflect management of undiagnosed mental health conditions or an improvement in overall emotional well-being.

Generalizability of results is limited, as the studied population is already enrolled in a digital health application which demonstrates an existing affinity for digital interactions. Participants were all commercially insured and the majority of them identified as white, thus future research is needed to determine whether this effect holds in a more diverse population.

## Conclusions

During the critical period of pregnancy (24, 30), digital mental health interventions have the potential to support mental health management, both by filling gaps in care and by educating and supporting pregnant people (14). As depression and anxiety disorders continue to account for a greater proportion of the world's disease burden (9, 31), this research provides an early indication that digital platforms with diverse offerings are a valuable addition to brick and mortar care and can educate and support pregnant people to help them manage anxiety and depression.

## Data Availability

The datasets presented in this article are not readily available because of privacy restrictions. Code is available upon request from the corresponding author. Requests to access the code should be directed to hannah.jahnke@mavenclinic.com.

## References

[1] Okagbue HI Adamu PI Bishop SA Oguntunde PE Opanuga AA Akhmetshin EM. Systematic review of prevalence of antepartum depression during the trimesters of pregnancy. Open Access Maced J Med Sci. (2019) 7:1555–60. 10.3889/oamjms.2019.270 31198472 PMC6542400

[2] Bauman BL Ko JY Cox S D’Angelo Mph DV Warner L Folger S Vital signs: postpartum depressive symptoms and provider discussions about perinatal depression—united States, 2018. MMWR Morb Mortal Wkly Rep. (2020) 69:575–81. 10.15585/mmwr.mm6919a2 32407302 PMC7238954

[3] Xu LL Li JQ Pu YQ Zhou C Feng SW Luo Q. Effect of prenatal depression during late pregnancy on maternal and neonatal outcomes. Clin Exp Obstet Gynecol. (2020) 47:681–6. 10.31083/j.ceog.2020.05.5398

[4] Margiotta C Gao J O’Neil S Vohra D Zivin K. The economic impact of untreated maternal mental health conditions in Texas. BMC Pregnancy Childbirth. (2022) 22:700. 10.1186/s12884-022-05001-6 36096759 PMC9464607

[5] Howard LM Khalifeh H. Perinatal mental health: a review of progress and challenges. World Psychiatry. (2020) 19:313–27. 10.1002/wps.20769 32931106 PMC7491613

[6] Sidebottom A Vacquier M LaRusso E Erickson D Hardeman R. Perinatal depression screening practices in a large health system: identifying current state and assessing opportunities to provide more equitable care. Arch Womens Ment Health. (2021) 24:133–44. 10.1007/s00737-020-01035-x 32372299 PMC7929950

[7] Novick G Womack JA Lewis J Stasko EC Rising SS Sadler LS Perceptions of barriers and facilitators during implementation of a Complex model of group prenatal care in six urban sites: implementing a complex model of group prenatal care. Res Nurs Health. (2015) 38:462–74. 10.1002/nur.21681 26340483 PMC4772136

[8] A growing psychiatrist shortage and an enormous demand for mental health services. *AAMC* https://www.aamc.org/news-insights/growing-psychiatrist-shortage-enormous-demand-mental-health-services [Accessed October 19, 2022].

[9] Whiteford HA Ferrari AJ Degenhardt L Feigin V Vos T. The global burden of mental, neurological and substance use disorders: an analysis from the global burden of disease study 2010. PLoS One. (2015) 10:e0116820. 10.1371/journal.pone.0116820 25658103 PMC4320057

[10] Andersson G Cuijpers P. Internet-based and other computerized psychological treatments for adult depression: a meta-analysis. Cogn Behav Ther. (2009) 38:196–205. 10.1080/16506070903318960 20183695

[11] Andrews G Basu A Guijpers P Craske MG McEvoy P English CL Computer therapy for the anxiety and depression disorders is effective, acceptable and practical health care_ an updated meta-analysis | elsevier enhanced reader. J Anxiety Disord. (2018) 55:70–8. 10.1016/j.janxdis.2018.01.001 29422409

[12] Krzyzaniak N Greenwood H Scott AM Peiris R Cardona M Clark J The effectiveness of telehealth versus face-to face interventions for anxiety disorders: a systematic review and meta-analysis. J Telemed Telecare. (2021) 0:1–12. 10.1177/1357633X211053738 34860613

[13] Kunkle S Yip M Hunt J Watson Ξ Udall D Arean P Association between care utilization and anxiety outcomes in an on-demand mental health system: retrospective observational study. JMIR Form Res. (2021) 5:e24662. 10.2196/24662 33496679 PMC7872836

[14] Hussain-Shamsy N Shah A Vigod SN Zaheer J Seto E. Mobile health for perinatal depression and anxiety: scoping review. J Med Internet Res. (2020) 22:e17011. 10.2196/17011 32281939 PMC7186872

[15] ..

[16] Din A Wilson R. Crosswalking ZIP codes to census geographies: geoprocessing the U.S. Department of housing & urban Development's ZIP code crosswalk files. Cityscape. (2020) 22:293–314. https://www.jstor.org/stable/26915499

[17] Department of Housing and Urban Development. HUD USPS ZIP Code Crosswalk Files. http://www.huduser.gov/portal/datasets/usps_crosswalk.htlml#data (2022)

[18] Song J South E Solomon S Wiebe D. Injustices in pandemic vulnerability: A spatial-statistical analysis of the CDC Social Vulnerability Index and COVID-19 outcomes in the U.S. (2021)2021.05.27.21257889. doi: 10.1101/2021.05.27.21257889).

[19] Greenland S Pearl J Robins JM. Causal diagrams for epidemiologic research. Epidemiology. (1999) 10:37–48. 10.1097/00001648-199901000-00008 9888278

[20] R Core Team. R: A language and environment for statistical computing. The R Foundation. (2021). https://www.r-project.org/

[21] Prescott MR Sagui-Henson SJ Chamberlain CEW Sweet CC Altman M. Real world effectiveness of digital mental health services during the COVID-19 pandemic. PLoS OnE. (2022) 17:e0272162. 10.1371/journal.pone.0272162 35980879 PMC9387818

[22] Zollars I Poirier TI Pailden J. Effects of mindfulness meditation on mindfulness, mental well-being, and perceived stress. Curr Pharm Teach Learn. (2019) 11:1022–8. 10.1016/j.cptl.2019.06.005 31685171

[23] Evans K Rennick-Egglestone S Cox S Kuipers Y Spiby H. Remotely delivered interventions to support women with symptoms of anxiety in pregnancy: mixed methods systematic review and meta-analysis. J Med Internet Res. (2022) 24:e28093. 10.2196/28093 35166688 PMC8889484

[24] Gawlik S Waldeier L Müller M Szabo A Sohn C Reck C. Subclinical depressive symptoms during pregnancy and birth outcome—a pilot study in a healthy German sample. Arch Womens Ment Health. (2013) 16:93–100. 10.1007/s00737-012-0320-0 23263748

[25] Walker MG Windrim C Ellul KN Kingdom JCP. Web-based education for placental complications of pregnancy. J Obstet Gynaecol Can. (2013) 35:334–9. 10.1016/S1701-2163(15)30961-0 23660041

[26] Chronister J Chou C-C Kwan K-LK Lawton M Silver K. The meaning of social support for persons with serious mental illness. Rehabil Psychol. (2015) 60:232–45. 10.1037/rep0000038 26009778 PMC4564308

[27] Naslund JA Aschbrenner KA Marsch LA Bartels SJ. The future of mental health care: peer-to-peer support and social media. Epidemiol Psychiatr Sci. (2016) 25:113–22. 10.1017/s2045796015001067 26744309 PMC4830464

[28] Lakey B Cohen JL Neely LC. Perceived support and relational effects in psychotherapy process constructs. J Couns Psychol. (2008) 55:209–20. 10.1037/0022-0167.55.2.209

[29] Tremain H McEnery C Fletcher K Murray G. The therapeutic alliance in digital mental health interventions for serious mental illnesses: narrative review. JMIR Ment Health. (2020) 7:e17204. 10.2196/17204 32763881 PMC7442952

[30] Stewart DE. Clinical practice. Depression during pregnancy. N Engl J Med. (2011) 365:1605–11. 10.1056/NEJMcp1102730 22029982

[31] Santomauro DF Mantilla Herrera AM Shadid J Zheng P Ashbaugh C Pigott DM Global prevalence and burden of depressive and anxiety disorders in 204 countries and territories in 2020 due to the COVID-19 pandemic. Lancet. (2021) 398:1700–12. 10.1016/S0140-6736(21)02143-7 34634250 PMC8500697

